# Assessing physical performance and physical activity in large population-based aging studies: home-based assessments or visits to the research center?

**DOI:** 10.1186/s12889-019-7869-8

**Published:** 2019-11-27

**Authors:** Erja Portegijs, Laura Karavirta, Milla Saajanaho, Timo Rantalainen, Taina Rantanen

**Affiliations:** Faculty of Sport and Health Sciences and Gerontology Research Center, University of Jyvaskyla, P.O. Box 35 (viv), Jyvaskyla, 40014 Finland

**Keywords:** Walking, Postural analyses, Aging, Functional impairment, Selection bias, Study design, Muscle strength, Exercise

## Abstract

**Background:**

The current study aims to compare correlations between a range of measures of physical performance and physical activity assessing the same underlying construct in different settings, that is, in a home versus a highly standardized setting of the research center or accelerometer recording. We also evaluated the selective attrition of participants related to these different settings and how selective attrition affects the associations between variables and indicators of health, functioning and overall activity.

**Methods:**

Cross-sectional analyses comprising population-based samples of people aged 75, 80, and 85 years living independently in Jyväskylä, Finland. The AGNES study protocol involved the following phases: 1) phone interview (*n* = 1886), 2) face-to-face at-home interview (*n* = 1018), 3) assessments in the research center (*n* = 910), and 4) accelerometry (*n* = 496). Phase 2 and 3 included walking and handgrip strength tests, and phase 4 a chest-worn and thigh-worn accelerometer estimating physical activity and assessing posture, respectively, for 3–10 days in free-living conditions.

**Results:**

Older people with poorer health and functioning more likely refrained from subsequent study phases, each requiring more effort or commitment from participants. Paired measures of walking speed (R = 0.69), handgrip strength (R = 0.85), time in physical activity of at least moderate intensity (R = 0.42), and time in upright posture (R = 0.30) assessed in different settings correlated with each other, and they correlated with indicators of health, functioning and overall activity. Associations were robust regardless of limitations in health and functioning, and low overall activity.

**Conclusions:**

Correlational analyses did not clearly reveal one superior setting for assessing physical performance or physical activity. Inclusion of older people with early declines in health, functioning and overall activity in studies on physical performance and physical activity is feasible in terms of study outcomes, but challenging for recruitment.

## Background

In aging research, managing the balance between standardized precision measures of physical performance and physical activity while minimizing bias due to selective drop-out may prove challenging [1, 2]. Health and functional limitations are frequently mentioned as reasons for not participating in research. To optimize the efficiency of data collection, flexible strategies are recommended [1, 3], while the efforts of participants need to be carefully weighed especially when aiming to include people with diverse health conditions in the study [1, 4]. The research setting, that is, the type and location of assessments may affect the participation rates [2].

Walking speed and handgrip strength are frequently used physical performance measures due to simplicity of the assessment and their predictive value for health and functioning in old age [5, 6]. Assessments of walking and muscle strength in the research center enable rigorous standardization and full control over the environment, but having to visit a research center may lead to increased participant burden and systematical attrition of people with poor health leading to biased results [2]. In order to include a wider range of people, walking speed and handgrip strength tests have also been incorporated in home assessment protocols [7, 8]. However, the environment and set-up for tests may not be completely standardized, potentially leading to a larger variation in testing conditions. On the other hand, testing someone in a familiar environment may be more relevant for daily life as one’s functioning at least partly depends on the environmental context [9].

Physical activity assessments have changed over the last decades from primarily self-report questionnaires to more objective measures. In recent years, technological advancements have enabled accelerometry-based physical activity assessments to be incorporated within large cohort studies in free-living environments [10–12]. These assessments require a participant to wear an accelerometer for multiple days in a row. Analyzing data from accelerometers lacks widely accepted standards and typically requires specific knowledge and skills, and devices may be costly. Thus, self-report questionnaires remain a frequently used alternative due to lower burden on participants and research staff [13, 14]. However, in older populations especially, validity of self-reported physical activity has been questioned due to potential problems in accurate recall and cognitive impairment [13, 15]. On the other hand, accelerometry that is based on intensity cut-points may not accurately reflect a person’s physical effort [16]. In older people especially, slow speed of movement coincides with increased energy costs for walking [11, 17]. Accelerometers attached to the thigh enable differentiation between postural positions, that is, sitting or lying versus upright or standing posture, and as such may pose an alternative approach to this challenge [18].

Results produced by questionnaires and measuring equipment that assess the same features of physical activity or performance in different settings are expected to correlate with each other. The current study aims to compare a range of measures of physical performance and physical activity assessing the same underlying construct in different settings, that is, in a home versus a highly standardized setting of the research center or accelerometer recording. Our goal is to help researchers to choose an appropriate test and setting for their future study. The current study also enables us to evaluate the selective attrition of participants related to the different settings and how this affects the associations between variables. Specifically, the aims were to study among older people 1) whether characteristics differ for participants of assessments conducted in different settings (home versus research center or accelerometer recordings), 2) associations between paired physical performance and physical activity measures assessed in different settings, and 3) whether assessment setting affected associations between these measures and indicators of health, functioning and overall activity. Finally, we also checked whether the associations varied markedly between people with and without limitations in health, functioning, and based on their overall activity.

## Methods

### Study design and participants

We present cross-sectional analyses of the observational ‘Active aging – resilience and external support as modifiers of the disablement outcome’ (AGNES) study. AGNES comprises three age cohorts (75, 80, and 85 years) of people living independently in the city of Jyväskylä, in Central Finland [19]. Our goal was to study 1000 people. Data were collected from September 2017 to December 2018. In late 2017 and early 2018, all people born in 1942 (interviewed predominantly from September 2017 to February 2018), 1938 (February–May 2018), and 1933 (April-Jun 2018) were invited to participate in the study. In 2018, additional sampling was conducted for those born in 1943, 1939 and 1934. Among the younger cohorts, we randomly selected approximately half of them and invited them to participate, while all of those in the oldest cohort were invited to obtain sufficient power for the planned analyses in the respective age-groups. The total population sample targeted was 2791 people. Exclusion criteria were not living independently in the recruitment area, and inability to communicate. Research methods have been reported previously in a protocol paper [19].

**Fig. 1:**
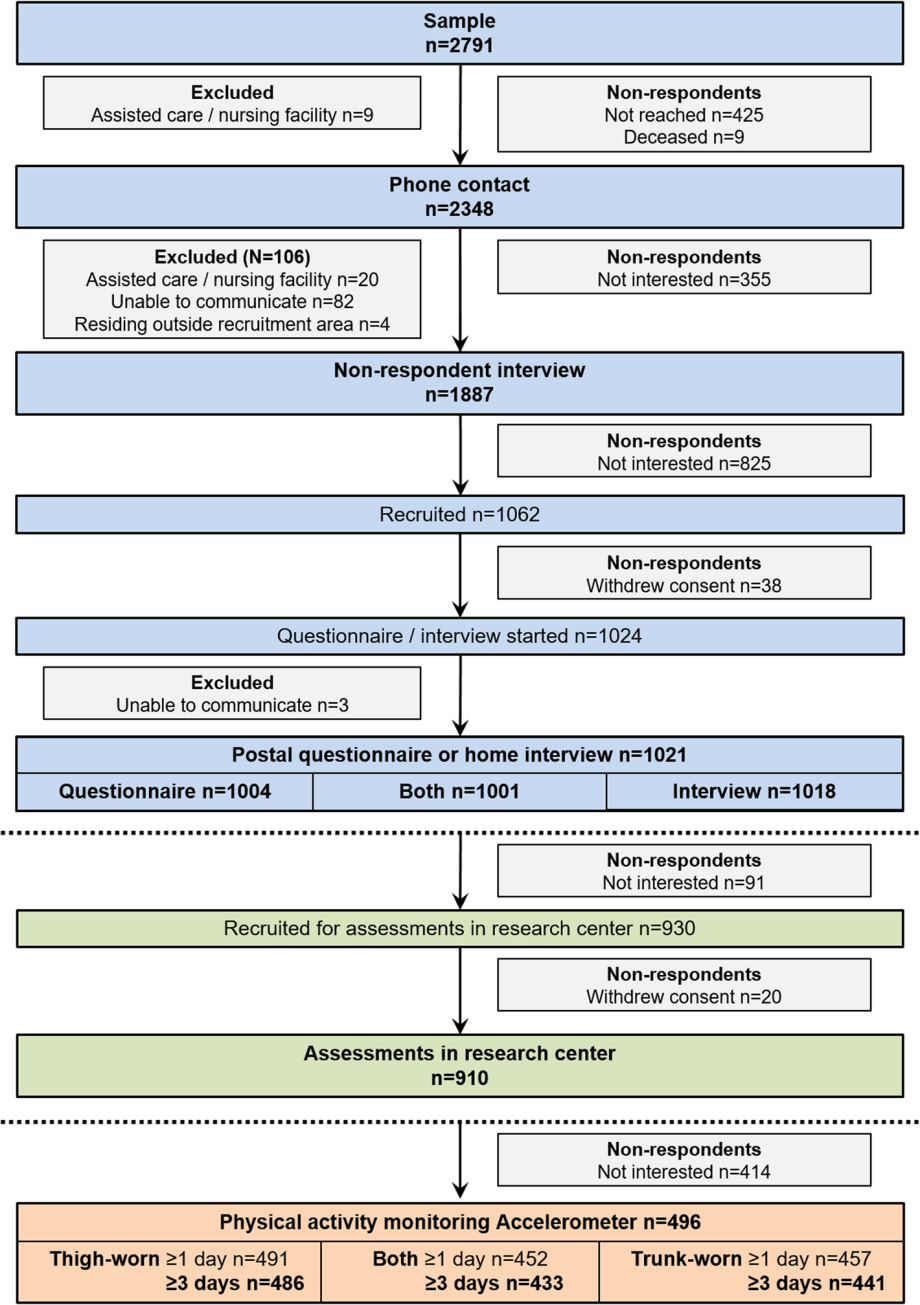
Flow chart of the study. At the end of the home interview, participants for the accelerometry study were recruited from those who agreed to participate in the assessments in the research center. However, the actual accelerometry data collection occurred prior to the assessments in the research center

Figure 1 displays a detailed flow chart including reasons for exclusion and non-participation at each contact. After an initial information letter and phone contact (*n* = 2348), a postal questionnaire was send to the willing participants and a face-to-face interview at the participants’ home, including some physical performance tests, was scheduled. Of the 1324 people who were contacted but refused to participate, 866 (65.4%) agreed to provide answers to a brief interview conducted over the phone. The postal questionnaire and home interview were completed by 1004 and 1018 participants, respectively. Home interview was conducted using computer assisted personal interviewing to minimize missing data. At the end of the home interview, the assessments in the research center were scheduled. Altogether, 910 participants attended the assessments in the research center. Transportation costs were compensated, and, if necessary, personal assistance for mobility was provided during the research center visit. Those consenting to the assessments in the research center were also offered the possibility to wear two accelerometers for the time between the home interview and the research center assessments. Accelerometry data was successfully collected for at least one day for 496 participants. As suggested earlier [3], we employed several strategies to facilitate retention in the study, including confirmation letters of time and place of assessments, sms reminders, and follow-up phone calls in case of no show. Interview times were scheduled flexibly at participants’ convenience, and, if requested, conducted at another location than the home. Participants were not offered any rewards for participation other than feedback on their health, functioning and overall activity. The ethical committee of the Central Finland Hospital district provided an ethical statement about AGNES on August 23, 2017. Participants signed an informed consent before the assessments.

### Main variables

#### Indicators of walking

*During the home interview,* walking speed was assessed over a distance of 3 m as part of the Short Physical Performance Battery [20]. A start and finish line were marked on the floor with tape and at least 60 cm was allowed for deceleration beyond the finish line. Walking time was measured using a hand-held stopwatch. *In the research center,* 10-m walking speed was assessed in the laboratory corridor and measured using photocells (Faculty of Sport and Health Sciences, University of Jyväskylä, Jyväskylä, Finland) [19]. Five meters was allowed for acceleration, and participants were asked to stop walking well past the finish line. In both tests, participants were instructed to walk at their habitual speed defined as the speed they would use when running errands. Participants wore walking shoes or sneakers. They were allowed to use a mobility device if needed; 26 did for assessments at home and 46 for the research center assessments, respectively. Walking speed (m/s) was calculated for the analyses.

#### Indicators of handgrip strength

At home and in the research center, maximal isometric handgrip strength was measured on the dominant side, defined as the side used to lift a heavy object onto a table. *During the home interview,* handgrip strength was measured with a hand-held adjustable dynamometer (Jamar Plus digital hand dynamometer, Patterson Medical, Cedarburg, WI, USA), and *in the research center*, using an adjustable dynamometer attached to a specific chair (Faculty of Sport and Health Sciences, University of Jyväskylä, Jyväskylä, Finland) [19]. The measurements were done in a seated position with the elbow flexed in an angle of approximately 90 degrees. After a practice trial, at least three maximal contractions were conducted until no further improvement occurred. The best test result was used in the analyses. In the assessment conducted at home, the inter-trial rest period was 30 s and the score was expressed in kilograms. This score was later transformed to units of Newton (N) by multiplying the obtained score with 9.80665. In the tests in the research center, a one-minute inter-trial rest period was used and the score was expressed in N.

#### Indicators of physical activity and posture

*Self-reported physical activity* was assessed in accordance with the Yale Physical Activity Survey for older adults [21]. Participants were asked how many times they performed vigorous physical activity and leisure walking for longer than 10 min during the past month, and the usual duration of a session. Daily minutes in walking and vigorous physical activity were approximated by recoding frequency responses to 0 ‘not at all’, 1 ‘1–3 times/month’, 2 ‘1-2 times/week, 4 ‘3-5 times/week’, and 6 ‘5+times/week’, and recoding duration responses to 20 ‘10–30 min’, 40 ‘30–50 min’, and 60 ‘60+ min’, and using these recoded frequency and duration scores in the following formula: (frequency*duration)/7. Subsequently, daily minutes in walking and vigorous physical activity were summed as an indicator of physical activity of at least moderate intensity. Additionally, participants were asked to estimate the duration of the time spent moving around and standing on an average day in the past month. Responses were converted to minutes as follows: 0 ‘not at all’, 30 ‘≤1 h/day’, 120 ‘1-3 h/day’, 240 ‘3–5 h/day’, 360 ‘5-7 h/day’, and 480 ‘7 + h/day’. Finally, we computed the estimated total time in upright posture by summing the estimated durations of the standing, moving around, walking, and vigorous physical activity.

In the *accelerometry-based physical activity assessment* participants were asked to wear two tri-axial accelerometers (both sampling continuously at 100 Hz, 13-bit±16 g, UKK RM42, UKK Terveyspalvelut Oy, Tampere, Finland, and 14-bit±16 g, eMotion Faros 180 including an additional electrocardiography (ECG) recorder not used for the current analyses, Bittium Corporation, Oulu, Finland) continuously during the time between the home interview and the assessments in the research center (typically seven to ten days) [19]. The accelerometers were attached to the thigh of the dominant leg and the sternum or diagonally on the left side of the chest under the breast to allow ECG recording and to ensure comfortable wear. The monitors were covered using transparent adhesive film for waterproofing. The eMotion Faros 180 sensor was swapped on the 3rd to 5th wear-day by the study staff at the participant’s home due to the expected battery life of four days. The tri-axial accelerations recorded by the two concurrently worn accelerometers were pre-processed identically as follows; the resultant (Euclidian norm) acceleration was first calculated for each sampling instant, and the mean amplitude deviation (MAD) [22] based on the resultant was subsequently calculated for non-overlapping 5 s epochs. The mean values of the X-, Y-, and Z-accelerations were also noted for the same epochs, and the 5 s epochs were assigned the real-time timestamp of the first data point included into a given epoch.

Physical activity minutes of at least moderate intensity were calculated from the trunk-worn sensor data by first calculating minute-by-minute means of the 5 s epoch MAD for each 24 h period from mid-night to mid-night and categorizing any minute with a mean MAD value from ≥0.091 g to < 0.414 g as moderate activity and ≥ 0.414 g as vigorous activity. These specific cut-points were validated in young adults to represent an intensity of at least three METs while walking on an indoor track [23]. The sum of the mean daily minutes in each intensity category was calculated. Moderate intensity and vigorous intensity activity minutes were subsequently pooled due to little data available for the latter.

The postural analyses utilize the thigh-worn accelerometer data. Sitting or lying and upright postures were evaluated by relying on having carefully mounted the thigh-worn accelerometer in a similar fashion on each participant. The accelerometer was mounted such that it should have read close to X = 0, Y = -1 and Y = 0 while the participant stood. Accordingly the vector [0, − 1, 0] was used as the reference orientation vector (¯R̅; $\overline{}$ indicates a vector) for subsequent posture evaluation, which was implemented following the approach presented by [24]. Subsequently an angle between the [*X*_*i*_ *Y*_*i*_ *Z*_*i*_] vector of a particular 5 s epoch (indicated by the letter i) and the reference orientation vector was calculated as

$$ {angle}_i= acos\left(\frac{\overline{R}}{\left\Vert \overline{R}\right\Vert}\bullet \frac{\left[{X}_i\kern0.5em {Y}_i\kern0.5em {Z}_i\right]}{\left\Vert \overline{\left[\begin{array}{ccc}{X}_i& {Y}_i& {Z}_i\end{array}\right]}\right\Vert}\right) $$

Where i = the index of the 5 s epoch of interest, ‖‖ indicates taking the norm, and ∙ indicates the dot product. The posture of each 5 s epoch was classified as lying or sitting if the *angle*_*i*_ was >π/4, or upright if the *angle*_*i*_ ≤π/4. Finally, the median posture category of each minute was used to represent the given minute of the 24 h-day, and the mean daily minutes in upright posture was reported as outcome in the present study.

### Other variables

#### Descriptive and anthropometric variables

Participants’ age and sex were derived from the population register. Based on previous research, physical performance and physical activity both may decline with age and may be lower for women than men [16, 25–27]. The dates of the assessments were used to compute the time interval between assessments and the season in which the home interview took place. Considering seasonal variation in physical activity [21] and the use of a retrospective physical activity questionnaire, the season at baseline was approximated as follows: winter January–March, spring April–June, summer July–September, autumn October–December). Other variables were collected using self-reports [19]. In the initial phone interview, people were asked about their living situation, that is, whether they lived in their home alone or together with someone else (e.g. spouse, children or other relatives, or someone else). Perceived financial situation, assessed using a single question with a 4-point likert-scale ranging from very good to poor, and educational level, defined as the total number of years in formal education, were used as indicators of socioeconomic status and assessed during the home interview. Body mass index was computed from body height (stadiometer) and body weight (electric scale Seca, Hamburg, Germany) measurements in the research center. Participants were categorized into underweight or normal weight (< 25.0), overweight (25.0–29.9), and obese (≥30.0) according to the WHO criteria [28]. The underweight and normal weight categories were merged due to low numbers in the first category. Research has shown that lower socioeconomic status and higher body mass index are associated with lower physical activity and poorer physical performance [26, 27].

#### Indicators of health and functioning

Indicators of health and functioning were assessed via self-reports [19]. Poorer health and poorer physical and cognitive functioning are associated with poorer physical performance [7] and lower levels of physical activity [25, 29]. During the home interview, self-rated health was assessed using a question on current general health with a five-point rating scale from one (very good) to five (very poor). On both occasions, participants were asked also to what extent their health or functional ability has prevented them from doing wanted activities during the previous four weeks. Responses ranged from not at all to much or very much on a five-point scale for the perceived limitations due to health or function variable. Cognitive function was assessed using the Mini-Mental State Examination [30] during the home interview. Perceived functional status was assessed in the postal survey by using a five-item questionnaire on basic Activities of Daily Living (ADL); feeding, rising from or lying down on a bed, dressing, bathing, and toileting. The number of items for which difficulty (some or a lot) or inability (with or without personal assistance) was reported was counted as indicator of ADL limitation. During the home interview, perceived walking difficulty over 500 m was asked using a validated question [31], and during the initial phone interview perceived outdoor walking difficulty was asked using a similar question. Response options on a five-point scale ranged from without difficulty to unable to manage even with help from another person. Use of different mobility device (e.g. walking stick, crutch, rollator) was assessed during the home interview and categorized as any mobility device vs. no mobility device.

#### Indicators of overall activity

Indicators of activity were assessed via self-reports [19]. Higher activity and mobility levels of older people have been associated with better physical performance and higher physical activity levels [7, 8, 32]. During the home interview, perceived neighborhood mobility was assessed as part of the Life-Space Assessment [33]. Participants reported how often they were moving in or beyond their neighborhood with response options of daily, 4–6 times a week, 1–3 times a week, and less than once a week. Accordingly, during the initial phone interview only, perceived outdoor mobility was assessed with a single question on frequency of going outdoors with the same response options. Habitual physical activity was assessed using a six-category single question during the home interview ranging from mostly sitting to participation in competitive sports. The 20-item version of Center for Epidemiologic Studies for Depression (CES-D) scale (score range 0–60) was used to assess depressive symptoms on a 4-point response scale from rarely or none of the time to most or all of the time during the previous week [34]. Depressive symptoms may reduce a person’s motivation to partake in activities, including physical activities, and as such lower physical activity and poorer physical performance levels are typically found among those experiencing more depressive symptoms [35, 36].

### Statistical analyses

Non-respondent analyses were conducted for the subsequent recruitment phases of the study which were participation in the home interview and/or postal survey, participation in the assessments in the research center, and participation in the accelerometry-based physical activity assessments. Chi-square, independent T-tests or Mann-Whitney U tests were used to test differences between the participants and non-respondents. Means and standard deviations or percentages were used to describe variables depending on their distribution. Group differences according to age cohort and sex were tested with independent T-tests or Mann-Whitney U tests and ANOVA or Kruskal-Wallis tests, respectively.

For the non-response analyses comparing older people declining to participate in any of the study phases and those participating in the home or postal questionnaire, variables were categorized as follows. Self-rated health was categorized into ‘good to excellent’, ‘fair’ and ‘poor or very poor’. The extent in which the person perceived limitations due to health or functional deficits was categorized into ‘not at all’, ‘to some extent’, and ‘much or very much’. Perceived outdoor walking difficulty was categorized into ‘a lot of difficulty or unable’, ‘some difficulty’, and ‘no difficulty’. Perceived outdoor mobility variable was categorized into ‘daily’, ‘4–6 times/week’, and ‘less frequently’.

For the subsequent non-response (home interview versus research center assessments, and research center assessments versus accelerometry) and subgroup analyses, all variables, except season, were dichotomized. Perceived financial situation responses were categorized as ‘good or very good’ versus ‘poor to fair’ financial situation. Self-rated health was categorized into ‘good to excellent’ versus ‘poor to fair’. The extent in which the person reported limitations due to health or functional deficits was categorized into ‘not at all’ versus ‘to some extent or very much’. Participants with a score of 24 or lower on the MMSE were considered to be at risk for cognitive impairment conforming current clinical guidelines and previous research [37]. ADL limitation counts were dichotomized as ‘no limitation’ versus ‘limitation in ≥1 items’. Perceived walking difficulty over 500 m was categorized as ‘no difficulty’ versus ‘at least some difficulty to unable even with help’. Perceived neighborhood mobility and perceived outdoor mobility was dichotomized as ‘daily’ versus ‘less frequently’. Based on the single question on habitual physical activity, participants reporting ‘light intensity activity or moderate intensity activity for ≤3 hr/week’ were compared with those reporting ‘>4h of moderate or vigorous intensity activity’ in accordance with previous studies [10]. In line with earlier studies, a CES-D score ≥ 16 was used to identify people with more depressive symptoms and who are at risk for clinical depression [35].

Correlations between paired physical performance and physical activity assessed in different settings were tested with Pearson correlation coefficients (R). We also checked whether these correlations varied markedly in subgroups of age, sex, cognitive impairment, ADL limitation, use of mobility device, neighborhood mobility, depressive symptoms, and season. In addition, partial correlation coefficients (R_p_), adjusting for age and sex, were calculated to study correlations between indicators of physical performance and physical activity measures and other indicators of health, functioning and overall activity to account for age- and sex-dependence of these associations. These analyses were conducted including the largest possible sample for whom the respective data were available. To determine the effect of potential bias due to selective attrition of participants in subsequent study phases, the correlational analyses were subsequently rerun including only those with both paired physical performance or physical activity measures available. Finally, we conducted sensitivity analyses to determine whether adding into the analyses those with only one to two days of accelerometer data would change the correlations found.

SPPS version 24 (IBM SPPS Statistics version 24, Armonk, New York, United States of America) was used for all statistical analyses and *p* < .050 was considered statistically significant.

## Results

### Non-respondent analyses

#### Overall study participation

Of the 2791 people approached, the overall participation rate in the current study was 36.6%, and it declined with age; 46.7% for 75-year-old; 34.7% for 80-year-old; and 27.0% for 85-year-old (*p* < .001). The recruitment rate was slightly higher for men (39.5%) than for women (34.7%; *p* = 0.001). About 15% (*n* = 425) of those approached could not be reached and about 4% (*n* = 119) was excluded due to not meeting the inclusion criteria. Of the non-respondents, 865 persons provided data for the non-respondent analyses in the initial phone interview. Most frequently reported reasons for not participating were lack of time, poor physical or cognitive health, and unwillingness to participate. In total, 8.7% of the phone interviews were answered by proxies (*n* = 164). Table 1 shows the non-respondent analyses stratified by age-cohort. Generally, those participating in the study reported better health and mobility than those of the same age not participating.

**Table 1:** Non-response analyses for the study phase conducted at home. Data were available for 857–1770 non-respondents and 1012–1021 participants depending on the variable. Results are stratified by age-cohort

		75 years		80 years		85 years		All	
		Non-respondent	Participant	Non-respondent	Participant	Non-respondent	Participant	Non-respondent	Participant
		% (n)	% (n)	% (n)	% (n)	% (n)	% (n)	% (n)	% (n)
Sex	Male	42.9 (224)	42.1 (193)	39.3 (249)	44.3 (149)	31.7 (195)	41.4 (94)**	37.7 (668)	42.8 (437)***
	Female	57.1 (298)	57.9 (265)	60.7 (384)	55.7 (187)	68.3 (420)	58.6 (133)	62.3 (1102)	57.2 (585)
Living situation	Alone	37.7 (95)	33.0 (151)	46.3 (151)	39.9 (134)	59.9 (170)	61.4 (135)	48.3 (416)	41.6 (420)*
	Together	62.3 (157)	67.0 (307)	53.7 (175)	60.1 (202)	40.1 (114)	38.6 (85)	51.7 (446)	58.6 (594)
Self-reported health	Good	38.1 (98)	53.5 (245)***	28.2 (92)	40.3 (135)***	17.7 (50)	32.7 (72)***	27.9 (240)	44.6 (452)***
	Fair	46.8 (118)	43.7 (200)	50.9 (166)	54.0 (181)	54.1 (153)	59.1 (130)	50.8 (437)	50.4 (511)
	Poor	14.3 (36)	2.8 (13)	20.9 (68)	5.7 (19)	28.3 (80)	8.2 (18)	21.4 (184)	4.9 (50)
Limitations due to health & function	Not at all	44.0 (111)	55.7 (255)***	32.6 (106)	46.0 (154)***	27.5 (77)	43.4 (95)***	34.3 (294)	49.8 (504)***
	To some extent	34.9 (88)	38.6 (177)	45.5 (148)	45.1 (151)	46.8 (131)	44.3 (97)	42.8 (367)	42.0 (425)
	Very much, much	21.0 (53)	5.7 (26)	21.8 (71)	9.0 (30)	25.7 (72)	12.3 (27)	22.9 (196)	8.2 (83)
Outdoor mobility	Daily	78.9 (198)	89.7 (411)**	73.0 (238)	84.8 (285)***	68.1 (192)	74.1 (163)**	73.1 (628)	84.7 (859)***
	4-6x per week	13.1 (33)	6.6 (30)	12.0 (39)	9.5 (32)	12.4 (35)	18.2 (40)	12.5 (107)	10.1 (102)
	Less frequently	8,0 (20)	3.7 (17)	15.0 (49)	5.7 (19)	19.5 (55)	7.7 (17)	14.4 (124)	5.2 (53)
Difficulty walking outdoors	No	57.1 (144)	76.4 (350)***	46.8 (152)	61.3 (206)***	36.2 (102)	50.5 (111)**	46.3 (398)	65.8 (667)***
	Some	27.4 (69)	18.6 (85)	36.9 (120)	33.0 (111)	40.1 (113)	37.3 (82)	35.2 (302)	27.4 (278)
	A lot / unable	15.5 (39)	5.0 (23)	16.3 (53)	5.7 (19)	23.8 (67)	12.3 (27)	18.5 (159)	6.8 (69)

Difference tested with Chi-square tests according to age cohort *p < .05, **p < .01, ***p < .001

#### Participation in the assessments in the research center

In total, 89.4% of those participating in the home interview also participated in the physical assessments in the research center (*n* = 910). Participation rates were higher for the younger cohorts (94.8% in 75-year-old, 86.9% in 80-year-old, and 80.7% in 85-year-old, *p* < .001). Men (89.7%) and women (88.5%) participated similarly (*p* = .586). Most frequent reasons for non-participation were lack of interest, lack of time, health-related issues or fatigue, and obligation to care for someone else. In total, 91.0% of the participants visited the research center 3–16 days after the home interview, half of them within nine days. Exceptionally long times between the home interview and the research center visit were due to illness and travels. Table 2 shows that participants attending the research center assessments generally reported better health and functioning, and higher overall activity than those choosing to participate in the home interview only.

**Table 2:** Comparison of participant characteristics in subsequent study phases: Those participating in the home interview only (*n* = 108) versus those taking part also in the subsequent physical assessments at the research center (*n* = 910); and those participating in the research center (*n* = 415) versus those also participating in the accelerometry-based physical activity assessments (*n* = 496)

	Home interview only	Research visit		Research visit only	Accelerometry	
	% (n) / Mean ± SD	% (n) / Mean ± SD	P	% (n) / Mean ± SD	% (n) / Mean ± SD	P
Age (75 years)	21.3 (23)	47.7 (434)	<.001 ^a^	44.3 (184)	50.5 (250)	.062 ^a^
(80 years)	38.9 (42)	32.1 (292)		32.3 (134)	31.9 (158)	
(85 years)	39.8 (43)	20.2 (184)		23.4 (97)	17.6 (87)	
Sex (Female)	60.2 (65)	56.9 (518)	.586 ^a^	53.5 (222)	59.8 (296)	.065 ^a^
Living situation (Alone)	51.4 (55)	40.0 (364)	.031 ^a^	44.3 (184)	36.5 (180)	.019 ^a^
Perceived financial status (Good-excellent)	58.3 (60)	60.5 (546)	.820 ^a^	57.5 (237)	62.9 (309)	.123 ^a^
Self-rated health (Good-excellent)	30.2 (32)	47.4 (431)	.001 ^a^	44.8 (186)	49.5 (245)	.180 ^a^
No limitations due to health & function	20.0 (21)	42.1 (383)	<.001 ^a^	41.9 (174)	42.2 (209)	.982 ^a^
No ADL limitations	77.3 (75)	88.5 (791)	.003 ^a^	86.1 (346)	90.4 (445)	.053 ^a^
No walking difficulty 500 m	57.6 (57)	79.8 (714)	<.001 ^a^	77.1 (314)	82.0 (400)	.089 ^a^
Mobility device (Yes)	43.0 (43)	21.5 (194)	<.001 ^a^	24.1 (99)	19.3 (95)	.096 ^a^
Physical activity level (Low PA < 3 h)	61.9 (60)	44.8 (401)	.002 ^a^	49.0 (199)	41.3 (202)	.025 ^a^
Neighborhood mobility (Daily)	46.1 (47)	62.2 (565)	.002 ^a^	62.8 (260)	61.7 (305)	.795 ^a^
Season (Winter)	17.6 (19)	22.2 (202)	.305 ^a^	20.0 (83)	24.0 (119)	.058 ^a^
(Spring)	28.7 (31)	21.2 (193)		24.3 (101)	18.6 (92)	
(Summer)	34.3 (37)	35.1 (319)		32.5 (135)	37.2 (184)	
(Autumn)	19.4 (21)	21.5 (196)		23.1 (96)	20.2 (100)	
Body Mass Index (Normal or underweight)	–	–	–	28.0 (116)	30.1 (149)	.524 ^a^
MMSE score	25.9 ± 3.0	27.3 ± 2.5	<.001 ^c^	27.1 ± 2.5	27.4 ± 2.4	.082 ^c^
CES-D score	9.5 ± 7.4	8.5 ± 7.1	.198 ^c^	9.5 ± 7.5	7.8 ± 6.6	<.001 ^c^
Years of education (years)	10.5 ± 3.9	11.7 ± 4.7	.025 ^c^	11.8 ± 5.1	11.6 ± 4.3	.956 ^c^
Max handgrip strength (N)	262.4 ± 93.8	310.3 ± 103.1	<.001 ^b^	303.0 ± 96.9	316.4 ± 107.7	.051 ^b^
Walking speed SPPB (m/s)	0.8 ± 0.3	1.0 ± 0.3	<.001 ^b^	0.9 ± 0.3	1.0 ± 0.3	.018 ^b^
Self-reported physical activity of at least moderate intensity (min/day)	30.4 ± 23.1	41.9 ± 27.6	<.001 ^c^	39.5 ± 27.1	43.9 ± 27.9	.006 ^c^
Self-reported upright posture (min/day)	303.6 ± 162.9	352.0 ± 148.6	<.001 ^c^	336.8 ± 144.1	364.7 ± 151.3	.004 ^c^

*ADL* Activities of Daily Living, *PA* Physical Activity, *MMSE* Mini-Mental State Examination, *CES-D* Center for Epidemiologic Studies for Depression

^a^ Chi-square test, ^b^ Independent T-test, ^c^ Mann-Whitney U test

#### Participation in the accelerometry-based physical activity assessments

In total, 54.5% of those participating in the physical assessments in the research center also participated in the accelerometry-based physical activity assessment (*n* = 496). Participation rates were somewhat higher for the younger age cohorts (57.6% in 75-year-old, 54.1% in 80-year-old, and 47.2% in 85-year-old, respectively, *p* = .062), and for women (57.1%) compared to men (50.8%) (*p* = .065), but statistical significance was not reached. Most frequent reasons for non-participation were lack of interest, wish to participate in water related activities, and travels. Of those who agreed to participate in the accelerometry-based physical activity assessments, 93.3% (*n* = 463) participants took both devices, 6.5% (*n* = 32) participants took the thigh-worn accelerometer only, and one participant took the trunk-worn accelerometer only. Data of four and seven participants were lost from the thigh-based or the trunk-based accelerometer, respectively, due to technical problems or discomfort wearing the device. Time spent in physical activity of at least moderate intensity (*p* = .522) and time spent in upright posture (*p* = .318) did not differ between those for whom ≥3 days of trunk- or thigh-worn accelerometer data was collected and those with 1–2 days of data available only (data not shown).

Table 2 compared participant characteristics of those participating in the accelerometry with those participating in the research center assessments only. Those participating in the accelerometry reported higher levels of physical activity during the home interview on self-reports than those not participating in the accelerometry (*p* ≤ .025). Furthermore, accelerometry participants reported more frequently living together with someone else, reported fewer depressive symptoms and had somewhat poorer physical functioning based on self-reports and physical performance tests at home compared to those participating in the assessments in the research center only.

### Correlation analyses

#### Indicators of walking

During the home interview walking speed was assessed in 995 participants (Table 3). Of those participating in the assessments in the research center, 892 participants had walking speed assessed at home and in the research center, eleven participants at home only, six participants in the research center only, and one not at all. For participants of both assessments, the mean walking speed was somewhat slower at home (0.97 ± 0.3 m/s) than in the research center (1.3 ± 0.2 m/s). Participants of the younger age groups (*p* < .001) and men (*p* ≤ .029) generally performed better on both walking tests (Table 6 in Appendix).

**Table 3:** Description and means (± standard deviation) of physical performance and physical activity (PA) scores in different settings, and Pearson correlation coefficients

	Home setting			Standardized setting			Correlation	
	Description	Total	Mean ± SD	Description	Total	Mean ± SD	N	R
Walking speed (m/s)	• Assessment: test during HI • Distance: 3 m and > 60 cm for deceleration • Device: stopwatch	995	0.96 ± 0.3	• Assessment: test in RC corridor • Distance: 10 m and 5 m for acceleration and deceleration • Device: photocells	898	1.3 ± 0.2	892	0.69***
Handgrip strength (N)	• Assessment: test during HI • Device: hand-held dynamometer • Protocol: 3–5 maximal contractions with 30s time interval	993	305.5 ± 103.1	• Assessment: test in RC • Device: chair-fixed dynamometer • Protocol: ≥3 maximal contractions with 1 min time interval	901	287.9 ± 100.0	888	0.85***
PA of at least moderate intensity (min/day)	• Assessment: questionnaire during HI • Definition: walking, and vigorous intensity PA • Data: month prior to HI	991	40.8 ± 27.4	• Assessment: trunk-worn accelerometer • Definition: moderate and vigorous intensity PA • Data: ≥3 full days between HI and RC assessments	441	28.5 ± 23.5	435	0.42***
Total upright posture (min/day)	• Assessment: questionnaire during HI • Definition: standing, moving around, walking, and vigorous intensity PA • Data: month prior to HI	988	347.3 ± 150.7	• Assessment: thigh-worn accelerometer • Definition: upright position • Data: ≥3 full days between HI and RC assessments	486	333.8 ± 103.0	480	0.30***

*HI* Home Interview, *RC* Research Center

***p < .001

The correlation between walking speed assessed at home and in the research center was R = 0.69 (Table 3). Walking speed assessed at home and in the research center showed comparable age- and sex adjusted partial correlations with all indicators of health, function and overall activity (Table 4), but there was a tendency of slightly higher correlation coefficients for walking speed assessed in the research center, which was underscored when those with both assessments available only were included in the analyses (data not shown).

**Table 4:** Partial correlation coefficients, adjusted for age and sex, between different indicators of health, functioning, and overall activity, and physical performance assessed at home or in the research center (RC), and physical activity (PA) assessed using questionnaire (Quest) or trunk- or thigh-worn accelerometers (acc.)

	Walking speed		Handgrip strength		PA of at least moderate intensity		Total upright posture	
	Home	RC	Home	RC	Quest	Trunk acc	Quest	Thigh acc
	*n* = 995	*n* = 898	*n* = 993	*n* = 901	*n* = 991	*n* = 441	*n* = 987	*n* = 485
Self-rated health	−0.27***	−0.35***	−0.19***	− 0.18***	− 0.32***	− 0.26***	− 0.23***	− 0.15**
Limited due to health & function	− 0.34***	−0.41***	− 0.19***	−0.16***	− 0.29***	−0.29***	− 0.21***	−0.19***
MMSE score	0.21***	0.11**	0.12***	0.11**	0.09**	0.04	0.11**	0.05
ADL limitation	−0.23***	−0.30***	−0.16***	− 0.11**	−0.14***	− 0.11**	−0.13***	− 0.07
Difficulty walking 500 m	−0.41***	− 0.45***	−0.19***	− 0.17***	−0.27***	− 0.31***	−0.26***	− 0.25***
Mobility device	−0.34***	− 0.37***	−0.17***	− 0.08*	−0.23***	− 0.24***	−0.20***	− 0.06
Physical activity – single question	0.42***	0.46***	0.22***	0.22***	0.49***	0.38***	0.43***	0.31***
Neighborhood mobility	0.32***	0.30***	0.15***	0.09**	0.38***	0.31***	0.27***	0.22***
CES-D score	−0.24***	−0.23***	−0.16***	− 0.14***	−0.18***	− 0.06	−0.18***	− 0.06
Body Mass Index	−0.20***	− 0.32***	0.04	0.10**	−0.24***	− 0.39***	−0.23***	− 0.40***
Days between home and RC visit	0.03	−0.02	−0.01	− 0.03	–	–	–	–
Monitor wear time	–	–	–	–	–	0.10*	–	0.03

*MMSE* Mini-Mental State Examination, *ADL* Activities of Daily Living, and *CES-D* Center for Epidemiologic Studies for Depression

Stronger correlations of paired home and lab result are bolded (difference in R ≥ 0.10), *p < .05, **p < .01, ***p < .001

#### Indicators of handgrip strength

Handgrip strength was assessed during the home interview in 993 participants (Table 3). Of those participating in the assessments in the research center, 888 participants had handgrip strength assessed at home and in the research center, seven participants at home only, thirteen participants in the research center only, and two not at all. Mean handgrip strength of those participating in both assessments was somewhat higher when assessed at home (310.5 ± 102.9 N) than at the research center (288.1 ± 99.5 N). For 9.3% of participants, the assessment side differed at home and in the research center. Participants of the younger age groups (*p* < .001) and men (p < .001) generally performed better on both handgrip strength tests (Table 6 in Appendix).

The correlation between handgrip strength assessed at home and in the research center was R = 0.85 (Table 3). When accounted for age and sex, handgrip strength assessed at home and in the research center correlated similarly with the indicators of health, functioning, and overall activity (Table 4), and any minor difference was mitigated after inclusion of those with paired handgrip strength assessments only (data not shown).

#### Self-reported and trunk accelerometry-based indicators of physical activity

During the home interview, 991 participants completed the questions about time in physical activity of at least moderate intensity. Of them, 892 attended the research center assessments, and this included all participants wearing the trunk-based accelerometer (*n* = 457; Table 3). For participants with both assessments, the mean time in physical activity of at least moderate intensity was 28.5 ± 23.6 min/day based on the trunk accelerometer and 44.2 ± 28.2 based on the questionnaire. There was a weak but statistically significant correlation between physical activity of at least moderate intensity and the number of days for which data was available (R = 0.10, *p* = .035). Participants of the oldest age group engaged less in physical activity of at least moderate intensity than those in the younger age groups based on the self-reports and trunk accelerometer (*p* < .001), but men reported more physical activity of at least moderate intensity than women based on the self-reports only (*p* = .013; Table 6 in Appendix).

The correlation between time in physical activity of at least moderate intensity from the questionnaire and the trunk-worn accelerometer was R = 0.42 (Table 3). Trunk-based and self-reported physical activity of at least moderate intensity correlated with indicators of health, functioning (except MMSE score for accelerometry only) and overall activity (except CES-D score for accelerometry only), when accounted for age and sex (Table 4). These correlations were practically similar for trunk accelerometer and questionnaire-based variables and did not markedly change when including only those with both measures available or when also participants with one to two days of accelerometer data were included in the analyses (data not shown).

#### Thigh accelerometry-based indicators of posture

The self-report questions on time in upright posture were completed by 988 participants in the home interview and 891 participants in the research center assessments (Table 3). Of those participating in the thigh-based accelerometry (*n* = 486), self-reports were missing for four participants. For those participating in both assessments, the mean time spend daily in upright posture was 333.7 ± 103.2 min/day based on the thigh accelerometer and 366.4 ± 152.1 min/day based on the questionnaire. The thigh-based postural indicator was not statistically significantly associated with the number of days for which data was available (*p* = .600). Participants of the oldest age group (*p* ≤ .025) and women (*p* ≤ .012) spend less time in upright posture based on thigh-based and self-reported postural indicators, respectively, than those in the younger age groups and men (Table 6 in Appendix).

The correlation between the self-reported and thigh accelerometer-based time in upright posture was R = 0.30 (Table 3). When accounting for age and sex, self-reports of upright posture time correlated with all indicators of health, functioning, and overall activity, but accelerometry-based upright posture time did not correlate with MMSE score, ADL limitation, mobility device, and CES-D score, respectively (Table 4). The somewhat stronger correlations of the questionnaire-based postural variable than the acceleromtery-based variable with indicators of health, functioning, and overall activity were leveled out, when including only those with both measures available in the analyses (data not shown). Additionally including participants with one to two days of accelerometer data into the analyses did not markedly change the results (data not shown).

#### Subgroup analyses

Correlations between paired physical performance and physical activity measures were similar for participants stratified by age group, use of mobility device and CESD score (Table 5). When participants were stratified by sex, correlations between physical activity of at least moderate intensity only was somewhat stronger for women than for men (R = 0.50 versus R = 0.31, respectively). When participants were stratified by MMSE score, correlations between paired physical performance and physical activity measures were relatively similar, or, if anything, for physical activity of at least moderate intensity only the association was marginally stronger for those with cognitive decline than for those without (R = 0.52 versus R = 0.40, respectively). When participants were stratified by ADL limitation, correlations between walking speed measures and time in physical activity of at least moderate intensity were somewhat stronger for those reporting ADL limitations than for those reporting no limitations (R = 0.81 versus R = 0.65; and R = 0.56 versus R = 0.40; respectively). Similarly, correlations between walking speed measures, time in physical activity of at least moderate intensity, and time in upright posture were marginally stronger for those reporting to move through the neighborhood less than daily than those moving through the neighborhood daily (R = 0.74 versus R = 0.62; R = 0.45 versus R = 0.32; and R = 0.44 versus R = 0.16, respectively). Finally, when stratified by season, paired measures of walking speed correlated somewhat more strongly with each other in summer than in winter (R = 0.78 vs. R = 0.63), and paired measures of time in physical activity of at least moderate intensity (R = 0.53 vs. R = 0.34) and time in upright posture (R = 0.41 vs. R = 0.24) correlated somewhat more strongly in summer than in autumn.

**Table 5:** Pearson correlation coefficients between paired assessments of physical performance and physical activity (PA) at home and in the research center according to different subgroups of participants

		Walking speed		Handgrip strength		PA of at least moderate intensity		Total upright posture	
		N	R	N	R	N	R	N	R
Age	75 years	430	0.63***	427	0.85***	225	0.41***	242	0.40***
	80 years	288	0.65***	285	0.85***	135	0.43***	153	0.15
	85 years	174	0.74***	176	0.86***	75	0.36**	84	0.29**
Sex	Male	385	0.68***	379	0.73***	176	0.31***	195	0.27***
	Female	507	0.69***	509	0.68***	259	0.50***	284	0.32***
MMSE score	25–30	786	0.68***	785	0.86***	385	0.40***	428	0.29***
	≤24	103	0.73***	100	0.79***	49	0.52***	50	0.35*
ADL limitation	No	780	0.65***	776	0.85***	390	0.40***	430	0.29***
	Yes	97	0.81***	99	0.86***	44	0.56***	47	0.37**
Mobility device	No	699	0.62***	702	0.84***	352	0.37***	383	0.31***
	Yes	186	0.71***	184	0.87***	80	0.45***	93	0.25*
Neighborhood mobility	Daily	554	0.62***	552	0.85***	269	0.32***	293	0.16*
	Less frequent	336	0.74***	335	0.86***	166	0.45***	185	0.44***
CESD score	0–15	745	0.67***	741	0.85***	375	0.42***	410	0.30***
	≥16	134	0.74***	136	0.89***	56	0.49***	63	0.25*
Season	Winter	200	0.63***	197	0.84***	103	0.46***	115	0.30**
	Spring	192	0.65***	189	0.85***	76	0.36**	90	0.28**
	Summer	192	0.78***	191	0.89***	89	0.53***	96	0.41***
	Autumn	308	0.68***	311	0.85***	174	0.34***	179	0.24**

*MMSE* Mini-Mental State Examination, *ADL* Activities of Daily Living, and *CES-D* Center for Epidemiologic Studies for Depression

*p < .05, **p < .01, ***p < .001

## Discussion

Participation rates in the different study phases show that older people experiencing health problems are less likely to participate in studies requiring more effort and commitment. This may potentially truncate the distribution of values. However, there was no clear evidence that this markedly compromised associations between measures of physical performance and physical activity, and indicators of health, functioning, and overall activity, especially when more standardized measures of physical performance and physical activity were used. Participation in the accelerometry study phase did not depend on health and functioning, but rather on depressive symptoms and general interest in physical activity. Furthermore, the findings of the current study suggest that it is feasible to study physical performance at home and in a research center even among those with limitations in health, functioning and low overall activity, including among those with early cognitive decline or depressive symptoms. Similarly, it is also feasible to assess time in physical activity of at least moderate intensity and time in upright posture using a questionnaire and accelerometry among people with a large range in health, functioning and overall activity. However, walking and physical activity may be more stable, and thus, more comparable between different measures, in summer than in other seasons. Consequently, study purpose and target population are important considerations for decisions of study setting and implementation strategy, as measures obtained in different settings generated similar results in the current study.

In line with previous research [1, 2, 4], the non-response analyses clearly showed that participants had better health and functioning than those declining to participate in subsequent study phases, requiring increasing effort and commitment from participants. Despite this selection bias, however, associations between physical performance and indicators of health, functioning and overall activity were similar regardless of the assessment setting (at home or in the research center). Especially for walking speed, the higher precision of the assessment in the research center seemed to compensate for the truncated values caused by selective attrition of participants from these assessments when compared to the full sample participating in the home interview. Non-participation in the accelerometry study phase did not clearly depend on participant effort in terms of health and functioning, rather depressive symptoms and general interest in physical activity played a role in the choice to participate, which are two acknowledged factors for physical activity participation in general [36]. Depressive symptoms may reduce a person’s motivation and increase an individual’s perceived effort [35]. Yet, in the current study, associations between the physical activity variables and indicators of health, functioning and overall activity were similar regardless of the assessment setting and any potential selection bias.

Isometric handgrip strength is considered a good general indicator of health and functioning regardless of whether it is assessed in the home setting or in more formal research center settings [6, 38], As expected, in the current research, handgrip strength assessed with similar devices in both settings correlated highly with each other, and correlations with other indicators of health, functioning and overall activity were virtually identical. Furthermore, associations between the home and the research center were unaffected by limitations in health, functioning, and overall activity. It seems that of the measures included in this study, handgrip strength was least prone to measurement error related to different assessment tools and protocols [38] and selection bias. Thus, handgrip strength measurement can be incorporated in different research settings without compromising its accuracy, but the measures used cannot be used interchangeably due to somewhat varying absolute values.

To date a large variety in methods (e.g. habitual and maximal walking speed) and distances (from about 2.4 m to ten or more meters) have been used to measure walking speed [5]. Regardless of the exact measurement protocol, walking speed is considered a good indicator of overall mobility function, and has been associated with many indicators of health, including institutionalization and mortality [39]. Recently, habitual walking speed has been proposed as a simple and safe measure to predict adverse outcomes in community-dwelling older people [5]. In the current study, habitual walking speed correlated with all indicators of health, functioning and overall activity regardless of whether it was assessed at home over a three meter distance or in the research center over a 10-m distance. Previously, it has been shown that, differences in measuring device (stopwatch vs. photocell) or distance should not affect the reliability of the measures [40]. The current results showed somewhat weaker correlations between paired walking speed for those with faster walking speed and better functioning, who in general are able to adapt their walking speed to varying life situations. For those with poorer functioning, stronger correlations or smaller variability in walking speed were found, which may relate to their overall slower walking speeds indicating reduced capacity [17]. Consequently, testing older people’s habitual walking speed seems feasible regardless of limitations in functioning, provided one can walk the distance safely. However, simple walking tests may still not accurately reflect real life situations requiring physical and mental flexibility to respond to environmental demands [41]. Furthermore, it has been shown that gait characteristics may change in formal testing situations compared to free-living environments [42, 43], which may explain why, in the current study, fewer participants used mobility devices during the home assessment. Physical activity was assessed in the free-living environment. Correlations between paired physical activity measures were somewhat stronger for those with limitations in functioning. Thus, our walking and physical activity results are in line with findings that older people with a reduced physical capacity may maintain their energy balance by walking more slowly and reducing their overall and physical activity [11, 44].

Increasingly, physical activity is measured using accelerometry [13, 45]. A common place of attaching the accelerometer is on the trunk at the hip. In the current study, the accelerometer was attached a bit higher on the trunk, due to the devices ability to also record ECG. Movement in the trunk is expected to be the same regardless of the exact attachment place, although some minor damping of the movement may occur. Self-reported physical activity minutes exceeded accelerometer-based activity minutes, which may be due to the absolute intensity cut-point being too high relative to the physical performance of the older adults, or due to the general difficulty to standardize the cut-point based analysis [11, 46]. Furthermore, physical activity intensity may be underestimated using trunk-worn accelerometers, especially in those walking more slowly [13] or performing other exercise modes besides walking on level ground [47]. Physical activity from self-reports, on the other hand, may be overestimated [48], but it is impossible to say which estimates better represent the physical activity of the participant as correlations between time in physical activity of at least moderate intensity and indicators of health, functioning and overall activity were similar regardless of assessment method.

Thus far, postural assessments from thigh-accelerometers have not been widely used. However, methods to establish posture and changes in posture have been validated before [24], and used even in frail hospital patients [18]. Postural assessment does not rely on intensity of the activity, but only determines whether a person is in an upright position or not. This may overcome problems related to assessing physical activity from slow movement accompanied by increased energy consumption typical in old age [16, 17], as most physical activity is conducted in upright position. Furthermore, low intensity physical activity according to traditional accelerometer definitions has shown to be beneficial for health and function in old age [49]. In the current study, correlations between the thigh-based upright posture time and indicators of health, functioning, and overall activity were relatively weak in general, and somewhat weaker than for the same measure derived from the questionnaire. Possibly, future research should combine indicators of intensity and posture to more accurately assess physical activity in the aging population.

With increasing age, the prevalence of cognitive impairment increases. Cognitive decline may affect an older adult’s willingness to participate in scientific studies and may pose challenges for the data collection [1, 15]. However, excluding older adults with cognitive declines, affects the generalizability of the study results [4]. In our study, we used ability to communicate with our research staff and independently living as study exclusion criteria. This means that some participants with early cognitive decline (MMSE score ≤ 24) were included in the study. In contrary to our expectations, in the current study, there were no indications of cognitively intact adults reporting their physical activity times more accurately than those with early cognitive decline. A study by Hauer et al. [29] also demonstrated that assessing physical activity through questionnaire is possible also among those with cognitive impairment, but they did find somewhat poorer associations between questionnaire-based and accelerometry-based physical activity for those with cognitive impairment, who were in a more advanced stage compared to the current study.

Study strengths are that this is a large population-based sample with large array of measures of physical performance and physical activity. The overall participation rate in the study was typical for current aging research, but the retention rate was very high within the different phases of this study. Despite employing several strategies to support study retention, the non-respondent analyses show, there was selective attrition from the start of the study and during each subsequent phase, which remains a common problem in aging research [2, 3]. There are several limitations to the study that limit generalizability of the results. The objective assessments of physical performance at home and those in the research center were not identical. While the handgrip strength measurements and walking tests have been validated some variation may exist due to differences in measurement protocols and algorithms used. Furthermore, it has been suggested that self-reported and accelerometer-based physical activity indicators may not measure the same thing, but may rather complement each other [12]. In the current study, the time periods for self-reported and accelerometer assessed physical activity did not overlap, which generates additional variation between the measures, especially in spring and autumn when external weather circumstances may vary more. In our study the selective drop-out may have been due to the rather intensive assessment protocols, with home interviews and the assessment in the research visit typically lasting about three hours. This may have scared off more fragile participants and those more prone to fatigue. Such drop-out may be at least partly avoided when only brief assessment sessions would have been proposed.

## Conclusions

Older people experiencing health problems are more likely to refuse to participate in studies requiring more effort and commitment. However, correlational analyses did not clearly identify one setting to be superior over the other for assessing physical performance or physical activity. Potentially, higher precision of assessments in standardized settings (research center or accelerometry) compensates for the somewhat truncated distribution of values due to selective attrition compared to more accessible home assessments. Therefore, no uniform conclusions can be drawn regarding optimal study setting and implementation strategy, but rather, decisions should be based on study purpose and target population. The findings support the feasibility of including in studies on physical activity and functioning also older people with limitations in health and functioning, including early cognitive decline.

## Data Availability

After completion of the study, data will be stored at the Finnish Social Science Data Archive without potential identifiers (open access). Until then, pseudonymized datasets are available to external collaborators upon agreement on the terms of data use and publication of results. To request the data please contact Professor Taina Rantanen (taina.rantanen@jyu.fi).

## Appendix

**Table 6:** Mean (± standard deviation) physical performance scores assessed at home (H) and in the research center (RC), and physical activity (PA) assessed by questionnaire (Q) and trunk- and thigh-based accelerometers (acc.), specified by age cohort and sex

	75-yr-old		80-yr-old		85-yr-old			Men		Women		
	N	M±SD	N	M±SD	N	M±SD	P	N	M±SD	N	M±SD	P
Walking speed (m/s)												
H	452	1.0±0.3	328	0.9±0.3	215	0.8±0.3	<.001	423	1.0±0.3	572	0.9±0.3	.001
RC	431	1.3±0.2	290	1.2±0.2	177	1.1±0.2	<.001	388	1.3±0.3	510	1.2±0.2	.029
Handgrip strength (N)												
H	451	328.0±103.7	325	304.8±103.9	217	260.2±84.4	<.001	419	388.0±87.7	574	245.5±65.2	<.001
RC	430	308.6±103.8	289	282.8±95.2	182	246.9±84.1	<.001	387	374.1±81.8	514	222.9±51.8	<.001
PA of at least moderate intensity (min/day) Q	448	43.8±28.1	325	41.1±26.8	218	33.9±25.8	<.001	421	43.3±28.5	570	38.9±26.5	.013
Trunk acc	228	32.1±24.0	137	27.9±22.9	76	18.6±20.3	<.001	177	29.5±24.0	264	27.8±23.2	.441
Total upright posture (min/day) Q	447	351.6±144.2	324	357.7±156.5	217	323.0±153.1	.004	419	334.3±154.4	569	356.9±147.3	.003
Thigh acc	245	326.9±103.7	155	350.4±101.5	85	323.3±101.6	.025	196	320.5±104.2	289	342.8±101.4	.012

Difference tested between age cohorts with ANOVA or Kruskal-Wallis tests and between sexes with independent T-tests or Mann-Whitney U tests

## Abbreviations

ADL: Activities of Daily Living
AGNES: Active aging – resilience and external support as modifiers of the disablement outcome
CES-D: Center for Epidemiologic Studies for Depression
MAD: Mean Amplitude Deviation
MMSE: Mini-Mental State Examination
N: Newton
